# Predictive Value of Spectral Computed Tomography Parameters in Esophageal Variceal Rupture and Bleeding in Cirrhosis

**DOI:** 10.5152/tjg.2023.21908

**Published:** 2023-04-01

**Authors:** Sunya Fu, Dawei Chen, Zhongwei Zhang, Ruiwei Shen

**Affiliations:** 1 Department of Radiology, Ningbo Medical Center Lihuili Hospital, Ningbo, China; 2 Department of Gastroenterology, Ningbo Medical Center Lihuili Hospital, Ningbo, China

**Keywords:** Cirrhosis, diagnostic effectiveness, hemorrhage, predictive value, spectral CT parameters

## Abstract

**Background:**

To evaluate the value of the spectral CT parameters in predicting the risk of esophageal variceal bleeding in cirrhosis with portal hypertension and to provide a reference for clinical diagnosis and treatment.

**Methods:**

Seventy-eight patients were divided into an esophageal variceal bleeding group and a non- esophageal variceal bleeding group. A comparison of variables including age, gender, platelet count, Child–Pugh classification, and spectral parameters between the 2 groups was done. Baseline model and spectral model were constructed with conventional parameters and conventional parameters coupled with spectral parameters, respectively. The 2 models were analyzed by the Receiver Operating Characteristic (ROC) curve.

**Results:**

The baseline model was established based on 4 conventional parameters and evaluated by ROC curve analysis. The spectral model was constructed based on the variables in the baseline combined with normalized iodine density in the liver parenchyma for the arterial phase, normalized iodine density in the liver parenchyma for the portal phase, normalized iodine density in the splenic parenchyma for the portal phase, diameter of the main portal vein, diameter of the splenic vein, and normalized iodine density of the left gastric vein. Normalized iodine density of the left gastric vein, normalized iodine density in the liver parenchyma for the portal phase, and Child–Pugh classification were the influencing factors of esophageal variceal bleeding in cirrhosis patients. The Area Under Curve (AUC) for the baseline and spectral models were compared (0.664 vs. 0.860) and the difference was found to be statistically significant (*P *< .001).

**Conclusions:**

The use of spectral CT parameters in consort with the conventional parameters can improve the diagnostic effectiveness of esophageal variceal bleeding in cirrhosis cases and screen for high-risk esophageal variceal bleeding patients. It may also provide an objective basis for the clinical prevention and treatment of esophageal variceal bleeding.

**Main Points**

- The normalized iodine density of spectral computed tomograghy (CT) can reflect the hemodynamic changes of cirrhosis.
- A logistic model integrating conventional parameters and spectral CT parameters is helpful in the clinical screening of high-risk esophageal variceal bleeding patients.
- The model in the second main pointabove has a good predictive capability of esophageal variceal bleeding in cirrhosis.

## Introduction

Esophageal varice is the most common complication of portal hypertension in cirrhosis with an incidence of 30%-40%.^1^ The 1-year mortality rate is 5% during the compensated cirrhosis stage and increases to 57% when cirrhosis progresses to the decompensated stage with esophageal variceal bleeding (EVB).^2^ The mortality rate within 6 weeks of acute EVB is as high as 20%.^3^ Once EVB occurs, the mortality rate is high and patient prognosis is severely hampered. Therefore, if the risk of EVB in patients with cirrhosis can be predicted in time, the survival time of patients with cirrhosis can be prolonged through clinically targeted medication or endoscopic intervention.

Spectral CT is noninvasive and reproducible, and the radiation dose is 35.9% lower than that of conventional enhanced CT.^4^ Spectral CT allows for the energy conversion of high (140 kVp) and low (80 kVp) to be resolved in a very short time (<0.5 ms), and for 101 single-energy images in the energy range of 40-140keV to be reconstructed.^5^ The imaging technique has a high degree of feature resolution and is one of the widely used iodine-based modalities.^6,7^ and can be used for multi-parameter quantitative analysis. Measured iodine levels on spectral CT directly reflect the iodine intake of various organs and indirectly the hemodynamic changes of the liver and spleen in cases of cirrhosis.^8^ As such, the purpose of this study was to evaluate the application of spectral CT parameters to predict the risk of EVB, reduce frequent endoscopic follow-up, and provide objective evidence for clinical prevention and treatment of EVB.

## Materials and Methods

### Study Population

About 78 patients diagnosed with cirrhosis via medical history, biochemical indicators, CT imaging, and ultrasound at our hospital from January 2019 to November 2020 were included in the study. Of these, 52 were male and 26 female; 28 were classified as Child–Pugh class A, 30 as class B, and 20 as class C; 50 were with hepatitis-associated cirrhosis, 11 alcoholic cirrhosis, 13 cases of cholestatic cirrhosis, and 4 of other causes. Inclusion criteria: patients underwent spectral CT and gastroscopy with satisfactory image quality and complete laboratory examination results. Exclusion criteria: (1) liver cirrhosis complicated with other tumors, portal embolism, and severe hepato-renal and spleen-renal shunt; (2) history of gastric, esophageal, or spleen surgery; (3) history of endoscopic esophageal variceal sclerotherapy, ligation, and other related treatments, as well as transjugular intrahepatic portal vena cava shunt surgery. First, patients underwent spectral CT then gastroscopy within 1 week. The criteria for EVB were as follows: active bleeding from a varice was observed under gastroscopy; varicose veins with “white nipples” and blood clots. Patients were then divided into an EVB group (40 cases) and a non-EVB group (38 cases). The platelet count of each patient was recorded. This study has been approved by the Ethics Committee of Ningbo Medical Center Lihuili Hospital (Approval NO. QT2020PJ058).

## Methods

*Dual-energy CT technique*. All CT scans were obtained using a 256-slice multidetector fast kV switching spectral CT (Revolution CT; GE Healthcare). Patients were placed in a supine position on the CT table and scanned in the craniocaudal direction, from diaphragmatic apex to the level of the lower margin of both kidneys. The contrast agent used was Iohydral (Yangtze River, nonionic, 350 mg I/mL) with a dose of 1.2-1.5 mL/kg, and an injection rate of 3 mL/s from the antecubital vein, followed by 30 mL saline injection. Current was set at 550 mA with fast-switch tube voltage (<0.5 ms) alternating between high (140 kVp) and low energy (80 kVp) voltages. For the arterial phase, automatic scan-triggering software was utilized (SmartPrep; GE Healthcare). Upon an abdominal aorta value of 140 HU, the scan was triggered and the portal phase scan was performed at 30 s after the arterial phase.

### Data Processing and Measurements

**Figure 1.**
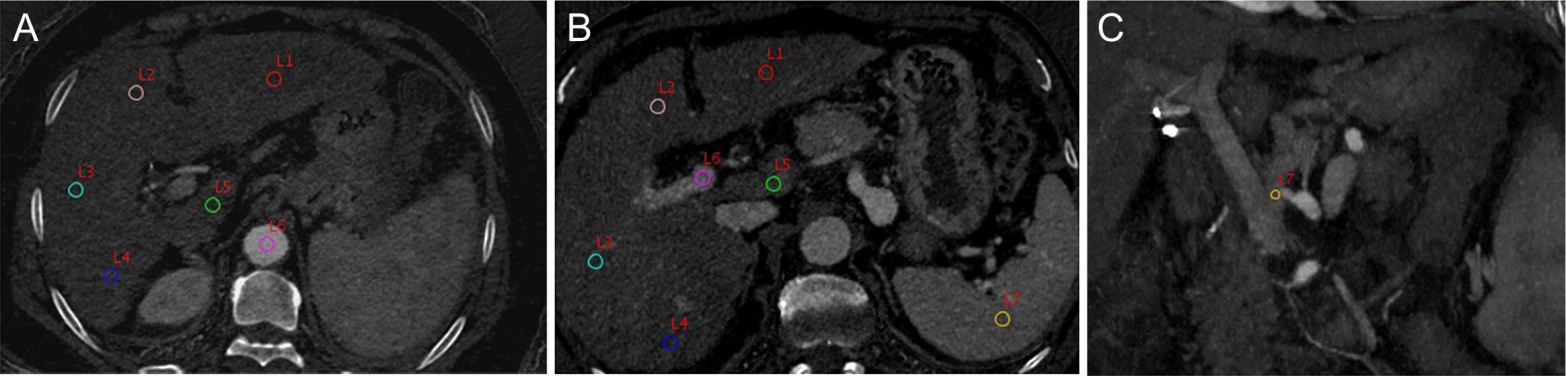
Spectral CT images (A) Axial ROI in hepatic artery phase (L1, L2, L3, L4, L5, and L6 represent iodine density (mg/mL) in the left lateral lobe, left medial lobe, right anterior lobe, right posterior lobe, caudate lobe, and abdominal aorta, respectively, in the hepatic artery phase). (B) Axial ROI in hepatic portal phase (L1, L2, L3, L4, L5, L6, and L7 represent iodine density in the left lateral lobe, left medial lobe, right anterior lobe, right posterior lobe, caudate lobe, main portal vein, and spleen parenchyma, respectively, in the hepatic portal phase). (C) Coronal ROI of the left gastric vein (L7 represent iodine density in the left gastric vein).

Spectral CT data were transferred to the GE AW 4.6 workstation, and the images were processed to obtain the iodine-based image. The ROI of the axial iodogram was (50 ± 2) mm^2^ at the conjuncture of the left and right branches of the portal vein, and iodine density (mg/mL) in each lobe of the liver was measured in the arterial phase (Figure 1A) and portal phase (Figure 1B). Iodine density in the abdominal aorta during the arterial phase, the main portal vein during the portal phase, the splenic parenchyma and the left gastric vein were measured on the same plane, and normalized iodine density was calculated. The diameter of portal trunk (at its widest) and the diameter of splenic vein (the maximum diameter within 2 cm from the point where splenic vein enters the portal vein) were measured under a single spectral image of 70 keV in the portal vein phase, and the normalized iodine density of each spectral parameter was calculated. All parameters were measured by 2 physicians, and the average of the 2 values was taken. The iodine density of the left gastric vein was measured on the iodine-based coronal image of the portal phase. The ROI area was 2/3 of the maximum diameter of the left gastric vein within 1 cm from its starting point (Figure 1C).

### Calculation Method of the Spectral Parameters

Mean hepatic iodine density (LID) = (left lateral lobe + left medial lobe + right anterior lobe + right posterior lobe + caudate lobe) iodine density/5

Normalized iodine density in the liver parenchyma for the arterial phase (NID_LAP_) = Mean hepatic iodine density/Abdominal aortic iodine density

Normalized iodine density in the liver parenchyma for the portal phase (NID_LVP_) = Mean hepatic iodine density/Hepatic portal iodine density

Normalized iodine density in the splenic parenchyma for the portal phase (NID_SVP_) = Splenic parenchyma iodine density in the portal phase/Hepatic portal iodine density

Normalized iodine density of left gastric vein (LGVI) = Left gastric vein iodine density/Hepatic portal iodine density

Diameter of the main portal vein (D_PV_) = Portal main diameter in 70keV portal venous phase

Diameter of splenic vein (D_SP_) = Splenic vein diameter in 70keV portal venous phase

Note: Ratio standardization of all quantitative measurements is carried out to eliminate differences in circulating blood velocity among individuals

### Statistical Analysis

The Statistical Package for Social Sciences (SPSS) version 22.0 (IBM Corp.; Armonk, NY, USA) and MedCalc 18.6 software were used for statistical analysis. The consistency between the 2 measures was assessed by the intraclass correlation coefficient (ICC) and an ICC > .75 was considered good. Then, the* k-S* test was conducted to determine whether the data conformed to normal distribution. Normally distributed data are reported as mean ± SD, non-normally distributed data percentiles *M *(*P*25, *P*75). The independent sample* t*-test was used for normally distributed data and the Mann-Whitney *U* test for the non-normally distributed. For statistically significant parameters, ROC curve was used to evaluate the diagnostic effectiveness, and the AUC was recorded to obtain the optimal threshold and corresponding sensitivity and specificity. Multivariate logistic regression was used to construct the baseline model and a spectral with conventional parameters and conventional parameters combined with spectral parameters, respectively. ROC curves were drawn to evaluate the diagnostic effectiveness of the 2 models and compared via the DeLong test. *P* < .05 was considered statistically significant.

## Results

### Conventional Parameters

There were no statistically significant differences in age, gender, and platelet count between the 2 groups; however there were significant differences with respect to Child–Pugh classification (*P *= .027) (Table 1).

### Logistic Regression Analysis and ROC Curves

Among the 4 parameters used in the baseline model, no statistically significant differences in age, gender, and platelet count were observed while significant differences were in Child–Pugh classification (Table 2). Baseline model: (*P*) = –0.673 + 0.816 (Child–Pugh classification), and the partial regression coefficient was 0.816. Child–Pugh classification was an influential factor for EVB in cirrhosis patients. The EVB predictive probability of the baseline model with conventional parameters was analyzed by ROC curve, and the AUC value was 0.664, the sensitivity was 77.5%, and the specificity was 50.0% (Table 3).

### Spectral CT Parameters

The ICC values of iodine density measured by the 2 observers were all greater than 0.75, indicating good stability of the parameters. For statistical analysis, the average of the spectral parameter values recorded by the 2 observers was taken. After the comparison of the spectral parameters between the 2 groups, it was found that there were statistical differences in NID_LVP_, NID_SVP__,_ and LGVI between the 2 groups. The NID_LVP_ in the EVB group was lower than that in the non-EVB group, LGVI and NID_SVP_ in the EVB group were higher than those in the non-EVB group. There were no statistically significant differences in NID_LAP_, D_PV__,_ and D_SP_ between the 2 groups (Table 4).

### Spectral Model

**Figure 2.**
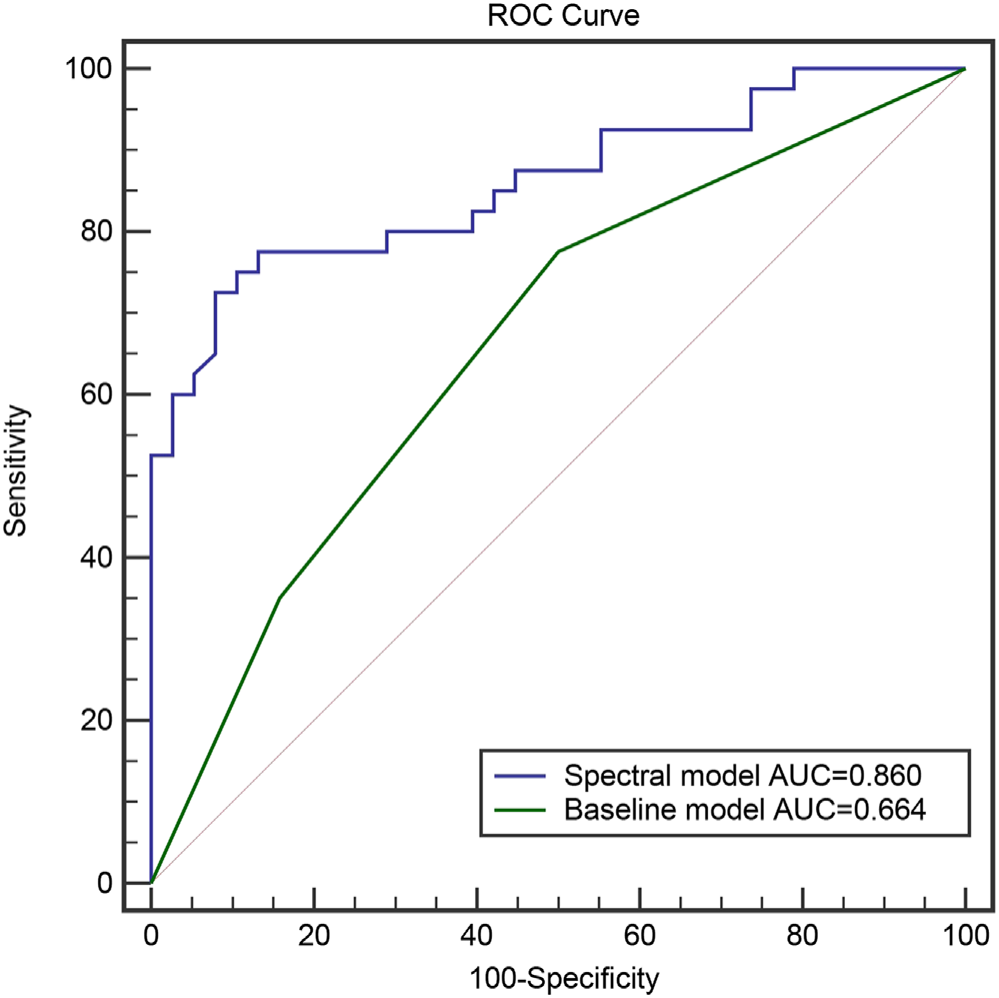
Comparative analysis of ROC curves of the 2 models.

In the spectral regression model, there were no statistically significant differences in NID_LAP_, D_PV_, D_SP__,_ and NID_SVP_ between the 2 groups. On the other hand, there were statistically significant differences in NID_LVP_, LGVI, and Child–Pugh classification between the 2 groups. Spectral model: (*P*) = –3.942 + 1.528 (LGVI) –1.852 (NID_LVP_) + 0.903 (Child–Pugh classification) (Table 5, AUC was 0.860, sensitivity 72.5%, and specificity 92.1%. The 2 models were analyzed by ROC curve and DeLong test, *P* < .001. The difference between the 2 groups was statistically significant (Table 6 and Figure 2).

## Discussion

Spectral CT is noninvasive and reproducible, with low radiation exposures and the ability to switch between energy levels instantaneously and obtain good resolution. This is applicable in screening high-risk EV and EVB patients with cirrhosis associated with portal hypertension. Dong et al^9^ have shown that the iodine density in the liver and spleen parenchyma on spectral CT can reflect the hemodynamic changes and severity of liver dysfunction in patients with cirrhosis. However, iodine density measured in their study was not standardized and was subject to individual differences in blood flow velocity. In our study, several hepatic and spleen spectral CT parameters were used for model construction, and the parameters were standardized.

The hepatic vein pressure gradient is the gold standard for the diagnosis of portal hypertension. Normal hepatic vein pressure gradient is no more than 5 mmHg. Studies have shown that when the hepatic vein pressure gradient is >12 mmHg, the risk of the onset of and recurrence of EVB in cirrhosis patients increases.^10^ However, the technique utilized in the particular study was limited by its invasion, difficulty, and high cost. The risk of EVB was predicted by observing the position, maximum diameter, and red color signs on the varicose veins under gastroscopy. However, gastroscopy is an invasive procedure, and the measurement of varicose vein diameter is highly subjective. In addition, patients with severe EV have the risk of iatrogenic bleeding, which patients do not accept.

Obstructed intrahepatic portal vein flow and increased peripheral circulation blood flow are the main factors in the formation of cirrhosis.^11^ In the early stage of cirrhosis, the portal venous blood flow decreases and activates the hepatic artery buffer response regulatory system.^12,13^ Adenosine is a vasodilator discharged from the periportal space of Mall into the hepatic sinusoids. It accumulates and activates receptors on the surface of the hepatic artery, triggering vasodilation. This regulatory mechanism can buffer the decrease of portal blood flow and maintain the total hepatic blood flow at a normal level. When portal blood flow further decreases during the decompensated stage of cirrhosis, compensation of hepatic arterial flow will fail. Consequently, the total hepatic flow will decline further, resulting in various complications.

In the results of this study, NID_LVP_ represents the situation of hepatic portal venous blood perfusion. The NID_LVP_ value in the EVB group was lower than that in the non-EVB group, and the decrease in portal blood flow in the EVB group was more apparent than that in the non-EVB group, which was in line with the mechanism of portal blood flow decline in cirrhosis. However, there was no significant difference in NID_LAP_ between the 2 groups, which reflected hepatic arterial blood perfusion. Perhaps when the hepatic portal blood flow declines, the hepatic arterial blood flow does not increase significantly, or the compensatory activity has been lost.

In this study, NID_SVP_ in the EVB group was higher than that in the non-EVB group. In cirrhosis, the whole body’s blood flow is in a state of high dynamic circulation, and the splenic venous blood flow increases from the normal 20%-30% to 60%-80%.^14^ Yin et al^15^ showed that the splenic venous blood flow and portal venous blood flow in the EVB group were significantly higher than those in the non-EVB group. The splenic venous blood flow ratio to portal blood flow was an independent risk factor for EVB. The left gastric vein, originating from the portal vein, typically flows to the liver. However, under portal hypertension, it changes from hepatic to bidirectional or is detached from the hepatic and reverses into the esophageal venous plexus. This reversal increases the blood flow of the lower esophageal venous plexus. When the continuously increasing blood flow exceeds the threshold that the vein can withstand, the esophageal vein ruptures, resulting in upper gastrointestinal bleeding. Hemodynamic changes in the left gastric vein during liver cirrhosis play a crucial role in predicting the risk of EVB.^16^ Our results showed that LGVI in the EVB group was higher than that in the non-EVB group, and the risk of EVB would increase when the left gastric venous blood flow increased.

Child–Pugh classification reflects the liver reserve function and the severity of liver dysfunction. The Child–Pugh scoring system was reported to be superior to other scoring systems in predicting EVB and its prognosis.^17^ We found no significant difference in age, gender, and platelet count between the EVB group and the non-EVB group. Only the difference in Child–Pugh classification was statistically significant. The baseline model constructed with conventional parameters to predict bleeding when analyzed by ROC curve analysis had an AUC of 0.664, sensitivity of 77.5%, and specificity of 50.0%. With the baseline model, Child–Pugh classification was determined to be a potential risk factor for EVB in liver cirrhosis. The model had a high sensitivity, low specificity, and low AUC values in predicting EVB risk. Child–Pugh classification did not show a simple linear relationship with the occurrence of EVB. We noted though that EVB did not occur in some patients classified as Child–Pugh class C, most likely due to the relative severity of liver dysfunction. However, due to the continuous opening of collateral circulation, the presence of spontaneous shunt leads to a decrease in portal pressure, a slight degree of EV, and a decrease in the incidence of EVB.^18^ Child–Pugh classification can only reflect the basic situation of liver function in liver cirrhosis. It cannot show the hemodynamic changes of the liver and spleen parenchymal, as such the AUC is not high. Considering this limitation, relaying solely on Child–Pugh classification to predict the risk of EVB is not reliable. To mitigate this, it is necessary to combine the conventional parameters with spectral CT parameters that can reflect the hemodynamic state to improve the diagnostic effectiveness of the prediction model.

Six spectral CT parameters, including NID_LAP_, NID_LVP_, D_PV_, D_SP_, NID_SVP_, and LGVI, were added to the baseline conventional parameters of age, gender, platelet count, and Child–Pugh classification. Logistic regression analysis showed that NID_LVP_, LGVI, and Child–Pugh classification were statistically significant factors. It has been previously reported that EV diameter, splenic vein diameter, ascites, iodine density of short gastric vein, and iodine density of splenic vein were independent predictors of EVB, the ROC curve analysis of the spectral model resulted in AUC higher than that of the baseline (0.839 vs. 0.809), and the difference between the 2 was statistically significant.^19^ In our study, the AUC value of the spectral model was 0.860 with a sensitivity of 72.5% and specificity of 92.1%. The LGVI and Child–Pugh classification were risk factors, and NID_LVP_ was a protective factor. When LGVI increased by 0.2, the potential bleeding risk increased by 3.6 times, and when NID_LVP_ increased by 0.2, the potential bleeding risk decreased by 84%. Upon comparison via the DeLong test, the predictive potential of the 2 models was significantly different (*P* < .001), the spectral model being superior. The diagnostic effectiveness of the baseline model was significantly improved when the spectral CT parameters were added (0.664 vs. 0.860).

There are notable limitations to our study. First of all, the patients in EVB group had upper gastrointestinal bleeding within 7 days. The hemodynamics of the liver and spleen may be affected by the different bleeding times during CT examination, and there is a lack of a strict and unified time standard. Second, the study was retrospective and the sample size was small contributing to selection bias. Finally, although the AUC for the spectral model of 0.860 indicates good diagnostic effectiveness, a large sample size prospective study is needed to validate the findings.

In conclusion, spectral CT imaging can reflect hemodynamic changes in the liver and spleen in cirrhosis patients. The NID_LVP_ and LGVI spectral CT parameters have a good correlation with the potential risk of EVB in cirrhosis. The spectral model established herein can improve the diagnostic effectiveness of EVB in cirrhosis, facilitate the screening of patients at high risk of EVB, provide an objective basis for clinical EVB prevention and treatment, as well as reduce the incidence and mortality of EVB in cirrhosis.

## Figures and Tables

**Table 1.** Comparison of Conventional Parameters between the 2 Groups

Classification	Non-EVB Group	EVB Group	Statistic (*t*/*z*/*χ^2^*)	*P*
Cases	38	40	_	_
Age (years)	57.97 ± 7.57	60.03 ± 10.13	−1.009^a^	.316
Gender (male/female)	27/11	25/15	0.641^b^	.423
Plt (×10^9^/L)	87.50 (51.00, 173.00)	64.00 (49.00, 89.25)	−1.631^a^	.103
Child–Pugh classification				
A	19 (50.0%)	9 (22.5%)	7.258^b^	.027
B	13 (34.2%)	17 (42.5%)		
C	6 (15.8%)	14 (35.0%)		

^a^ *t*-Value.

^b^ *χ^2^*-Value.

EVB, esophageal variceal bleeding; Plt, platelet.

**Table 2.** Baseline Model Analysis

Parameter	*B*	SE	Wald Value	*P*	OR
Child–Pugh classification	0.816	0.317	6.649	.010	2.262
Constant	−0.673	0.364	3.409	.065	0.510

**Table 3.** ROC Curve Analysis of Conventional Parameters between the 2 Groups

Parameter	AUC	Cutoff level	Sensitivity (%)	Specificity (%)
Child–Pugh classification	0.664	0.338	77.5	50.0

**Table 4.** Comparison of Spectral CT Parameters between the 2 Groups

Classification	Non-EVB group	EVB group	Statistics (*t*/*z*)	*P*
N	38	40	_	_
NID_LAP_	0.06 (0.05, 0.07)	0.05 (0.04, 0.07)	−1.757^b^	.079
NID_LVP_	0.44 ± 0.07	0.39 ± 0.38	3.302^a^	.001
NID_SVP_	0.59 ± 0.03	0.61 ± 0.05	−2.159^a^	.035
D_pv_ (mm)	15.35 ± 0.83	15.71 ± 0.97	−1.728^a^	.088
D_SP_ (mm)	10.22 ± 0.97	10.61 ± 1.09	−1.649^a^	.103
LGVI	0.80 (0.75, 0.92)	0.98 (0.86, 1.14)	−4.349^b^	<.001
Child–Pugh classification				
A	19 (50.0%)	9 (22.5%)	7.258^c^	.027
B	13 (34.2%)	17 (42.5%)		
C	6 (15.8%)	14 (35.0%)		

^a^ *t*-Value.

^b^ *z*-Value.

^c^ *χ^2^*-value.

D_PV_, diameter of the main portal vein; D_SP_, diameter of the splenic vein; EVB, esophageal variceal bleeding; LGVI, normalized iodine density of the left gastric vein; NID_LAP_, normalized iodine density in the liver parenchyma for the arterial phase; NID_LVP_, normalized iodine density in the liver parenchyma for the portal phase; NID_SVP_, normalized iodine density in the splenic parenchyma for the portal phase.

**Table 5.** Logistic Analysis after Adding Spectral CT Parameters

Parameter	*B*	SE	Wald Value	*P*	OR
LGVI	1.528	0.418	13.362	.000	4.611
NID_LVP_	−1.852	0.897	4.263	.039	0.157
Child–Pugh classification	0.903	0.388	5.426	.020	2.468
Constant	−3.942	2.684	2.158	.142	0.019

LGVI, normalized iodine density of the left gastric vein; NID_LVP_, Normalized iodine density in the liver parenchyma for the portal phase.

**Table 6.** ROC Curve Analysis of the 2 Logistic Regression Models

Group	AUC	Cutoff level	Sensitivity (%)	Specificity (%)
Baseline model	0.664	0.338	77.5	50.0
Spectral model	0.860	0.666	72.5	92.1
